# Design and manufacture of a scaled railway track with mechanically variable geometry

**DOI:** 10.1038/s41598-022-12554-1

**Published:** 2022-05-23

**Authors:** Rosario Chamorro, Javier F. Aceituno, Pedro Urda, Enrique del Pozo, José L. Escalona

**Affiliations:** 1 Departamento de Ingeniería Mecánica y Fabricación, Universidad de Sevilla, 41092 Sevilla, Spain; 2 Departamento de Ingeniería Mecánica y Minera, Universidad de Jaén, Jaén, Spain; 3 MC2 Ingeniería y Sistemas S.L., Sevilla, Spain

**Keywords:** Engineering, Mechanical engineering

## Abstract

The objective of this article is to present the design and manufacture of a scaled railroad track to be used as a laboratory track for the study of different railway applications. It could be a guideline for future laboratory railroad tracks. The ideal concept was based on possible future studies and, according to them, design requirements have been specified. The main characteristic of the track is that its geometry can be mechanically modified and irregularities can be introduced under controlled conditions in any kind of track sections: straight, curved and transition ones. Finally, the current installed track is shown and the performed quality controls are described.

## Introduction

In the last 7 decades, the operational train speed has greatly increased with the development of high-speed trains from countries such as Japan (Shinkansen), France (TGV), Germany (ICE), China (CRH) and Spain (AVE). An example of this improvement is the speed record of 159.67 m/s (574.80 km/h) reached by TGV Alstom in 2007^1^.

When studying railway dynamics, the behaviour of railway vehicles can be simulated in a virtual environment using commercial programmes. A list of the main available programmes can be found in^2^. Several benchmarks^3–7^ have allowed to compare agreement between different codes giving confidence on results, acknowledging that no analytical solution is known. Although computational models are very useful, it is important to experimentally validate the numerical results to ensure that the model is sufficiently accurate in the simulation of reality. To this end, validation requires experimental data from real vehicles to be obtained. Due to the difficulty of having access to this type of data, laboratory solutions exist for certain studies. Two examples of these laboratory solutions are full and reduced-scale roller rigs, and full and scaled vehicle-track systems. A state of the art of the scaled roller rigs can be found in^8–10^. Each roller rig design is unique and strongly depends on the goals of experiments, as it is highlighted in^11^. In relation to full scale vehicle- track systems, there is an important facility in USA, the Transportation Technology Center, in Pueblo, Colorado, that has been testing vehicles and track components since 1970s^12^. New facilities will be operational within 2022^13^. Another full scale system can be found in Japan, MIHARA test center^14^. In relation to scaled vehicle-track systems, if the main purpose is to obtain results extrapolated to a full-scale system, the problem of scale correlation and similarity laws implies that experimental results in the scaled track cannot be directly extrapolated to results in a full-scale track^15,16^. It allows, however, the validation of computational models as its main advantage. In this sense, roller rigs have proven to be an effective experimental tool for the study of wheel-rail contact and other issues in railway vehicle dynamics^17^. However, roller rigs present certain disadvantages versus the use of test tracks due to some differences described as follows:

1. The curvature of the roller alters the geometry of the contact area between wheel and roller, the contact patch being shorter in the direction of motion. This difference will influence the magnitude of the creep forces^8,18,19^.
2. The lateral and vertical structural stiffness of rollers may be different from the track stiffness.
3. Wear patterns: in tracks, testing wear is more uniformly distributed whilst in a roller rig it is more concentrated in the roller surface^8^.
4. Introducing track irregularities in roller rigs is complex and tedious since it implies an additional source of vibrations to the roller. Some examples of roller rigs that simulate track irregularities in curved tracks can be found in^10,20^, as Chengdu and Japan (RTRI) roller rig.
5. It is complex for a roller rig to simulate a transition curve. Owing to this, studies are limited to straight or constant-radius curved tracks.

To the best of the authors’ knowledge, there is very little bibliography describing construction for research purpose. The University of Tokyo has the Chiba Experiment Station, IIS, with a 25 m length scaled track (1/10) formed by the following segments: a straight section, transition curve, curved track (*R* = 3.3 m), transition curve and a straight one. In this track, whose description can be found in^21–23^, the transition curves are sinusoidal. Also, in^24,25^ it is explained the construction of a testbed for the development and testing of active steering systems of railway vehicles, and it includes a 1/5 scaled track model with no cant angle and two straight tracks (6.41 m length each one) connecting a curved track with *R* = 20 m and 14.30 m length. In^26^, a 1/5 scaled wheel-rail test rig is used. It is a beam mounted on a central pivot with a single wheel at one end of the beam, and a circular track with a diameter of 2.5 m. In^27–29^, a rail track assembly is described. It includes different subassemblies as the components of a railway track structure, which is bended around a central pivot to provide a circular rail ring with a diameter of 4 m. Another example of a scaled track is that of Stapleford in Leicestershire (UK), which is used for training purposes by the railway division of the Institution of Mechanical Engineers through the *Railway* *Challenge* *Competition*. This competition is focused on the knowledge of the complete cycle of the design and manufacture of a prototype supported by academic and industry mentors. Participants should design and build a scaled locomotive that can travel on a 0.26 m (10 1/4”) gauge track following strict rules and specified technical details. The locomotives are tested on the aforementioned track.

This article describes the design and manufacture of a scaled railroad track that allows its geometry to be mechanically modified. The rails have sufficient degrees of freedom to introduce, under controlled conditions, the 4 types of irregularities commonly measured in conventional tracks: alignment, gauge deviation, cross-level and vertical profile^30–32^. This track allows the study of the dynamic behaviour of different railway vehicles under irregularities in straight, curved and transition track sections. There are experimental facilities that allow to study the dynamic response of a vehicle to different track irregularities but the scaled track described in this paper allows to change the irregularities in a mechanical and controlled way. A railway track in which the geometry can be mechanically modified to reproduce irregularities is novel and, as far as the authors know, unique in the world. The possibility of changing the geometry of the track allows a wide range of possibilities, not only for the introduction of the 4 well-known track irregularities but also, for instance, the cant angle modification at curves. In addition, it is a system that is always accessible to conduct low-cost experimental tests, and it has the advantages of a track versus a roller rig, although due to the scaling geometry, a strategy on similarity laws should be followed to extrapolate results to conventional tracks, as shown in^33,34^. The main objective is to study railway dynamics experimentally, to validate numerical methods and to develop new control units that can be extrapolated or used in conventional rail systems. The track is not designed to directly extrapolate the dynamics effects obtained at the scaled size to the full-scaled one. Mass and stiffness of the track have not been scaled.

The paper is divided as follows: ‘Design requirements” section which details the design requirements considered for the purpose of the present and future research; “Final design and manufacture” section shows the final design and manufacture that meet the required specifications and “Quality controls performed on the scaled track” section explains the quality controls carried out to ensure that the specifications are met. Finally, “Summary and conclusions” section gathers a summary of this work and conclusions.

## Design requirements

In this section the design requirements are specified. Some of the requirements are justified for research purposes, while others are required to verify the proper construction of the track.

As already mentioned, the main characteristic of the scaled track is that its geometry can be modified mechanically, and irregularities can be entered in a controlled manner. Some of the studies that this track allows are:

1. To develop on-board systems capable of analysing the vehicles’ dynamic responses to track irregularities. The track allows the experimental measurement of the dynamics of scaled rail vehicles, as the one presented in^35^ following similarity laws. Although the mechanically scaled introduced irregularities are related to the EN13848^32^ standard wavelength ranges of *D*1 ($\lambda$ $\in$ [3,25] m), *D*2 ($\lambda$ $\in$ [25,70] m) and *D*3 ($\lambda$ $\in$ [70,150] m), valid for alignment, track gauge, vertical profile and cross-level, it could also be used for the analysis of the short-wavelength irregularities below 1-m wavelength (such as corrugation). In the latter case those short-wavelength irregularities can only be introduced permanently. To this end, the purpose of knowing the existing irregularities is to verify that the developed methods to monitor the track geometry are capable of capturing the known irregularities. Some studies have already been carried out and can be found in^36^.
2. To develop systems for measuring the contact forces in the wheel-rail interface. Currently, the measurement of the contact forces with dynamometric wheelsets is the most precise method. However, calibration of full-scale dynamometric wheelsets is a complex and expensive process, and under certain scenarios might not be entirely accurate^37^. This process is highly simplified in a scaled track. Some studies have been carried out in the scaled track described in this paper measuring vertical, lateral and longitudinal forces as shown in^38^. The contact forces measured in^38^ and validated with computational models cannot be directly extrapolated to the contact forces obtained in a full-scale system due to similarity laws, but the measurement techniques can be extended to real vehicles following an equivalent methodology.
3. Effect on vehicle dynamics of the change in track stiffness as it occurs when there is a bridge.

The location of the rail track is the roof of the Higher Technical School of Engineering of the University of Seville. Due to this location, there have been limitations on the layout decision and certain restrictions that have become design specifications.

The main design requirements for research purposes are the following:

1. The track geometry should have the kind of sections found in conventional tracks: straight, constant curvature and transition sections that connect sections of different curvature. Ascending and descending slopes should also be included in the geometry.
2. Each rail should move independently in vertical and lateral direction (2 degrees of freedom for each rail in the cross-section plane). The possibility of lateral movement will allow the track gauge deviation and irregularities in the alignment to be reproduced. The vertical displacement will allow the irregularities of cross-level and vertical profile to be reproduced and vertical slopes to be modified.
3. Possibility to generate an arbitrary cant angle is required.
4. The method of varying the track geometry should be easily applied.
5. Rails should be discretely supported by sleepers with elastomeric material as in most conventional tracks.
6. Each rail section should be as long as possible to reduce the number of discontinuities in the final track.
7. Vertical and lateral step in the rail joints (minimum mismatch of position permitted between the sections in contact with consecutive sections of rail) should not affect the dynamics of the railway vehicle much when passing through consecutive rail sections. Welded connections are not allowed to permit easy substitution of rails (i.e. worn rails, rails with corrugated surface, rails with different profiles, etc.).
8. Separation between consecutive rail sections is not allowed to minimize the influence on the dynamics of the railway vehicle as it passes through the joints, except for the consideration of expansion joints that absorb the length variation of the rails due to temperature effects.
9. The track should include a bridge-like structure. The bridge allows the vehicles to experience a change in the track stiffness with respect to the general track base structure.
10. The rails must not be permanently deformed by the usual loads they are subjected to. Low-weight railway vehicles (approximately 50 kg) will be tested on the track.

The design requirements due to the location and available space are the following:

1. 5-inch track gauge (127 mm). This implies a length scaling factor with respect to a standard 1435 mm track gauge of 1/11.3.
2. Impact on the roof where the track is installed should be minimal. In this sense, roof should not be perforated.
3. The structure should guarantee sufficient stability to avoid the displacement of the track and should allow the evacuation of rainwater from the roof of the building.
4. The material of the support mechanisms should be adequate to avoid corrosion, taking into account that the track will be installed outdoors.

## Final design and manufacture

In this section, the track final design will be described. The design of three parts in the track are described next: infrastructure, support mechanisms and rails.

### Infrastructure

With the specifications required for the infrastructure, one of the most proper solutions would be the use of a concrete foundation that simulates a slab track^39^. However, due to the limitations at the location of the track (the roof of an Engineering School and the restrictions established by the University of Seville), metallic tables simply supported on the roof are considered.

On the tables, constituted by a structure of electro-welded steel tubes and equipped with adjustable feet, a 6 mm thick steel plate cover is screewed with a series of perforations intended to receive the fastening screws of the support mechanisms that will serve as the support for the rails. Both the structures of the tables and their surfaces are built in hot-dip galvanised steel according to UNE-EN-ISO 1461: 2010^40^, to provide them with adequate protection against corrosion. There are 47 tables along the circuit. The size of each table has been limited to a maximum length of 2 m to facilitate individual handling. Each structure is bolted with the adjoining ones, forming a continuous chain to assure rigidity and stability. Due to the drainage slopes of the roof, the tables supports are adjustable which allows to compensate differences in elevation and inclination. The average height of the tables is 0.7 m and the higher level of the rail runs at a height of 0.825 m. The advantages offered by this system compared to a system such as conventional ballast or slab scaled tracks are the following:

1. The impact on the roof of the building is minimal. A structure similar to the conventional ones would affect the drainage of rainwater which would increase the risk of leaks in the building.
2. A great reduction on the dead load added to the roof with respect to a concrete foundation is achieved. In this sense, a concrete foundation would add around 630 kg/m versus the 50 kg/m of the proposed infrastructure.
3. The proposed system allows a positioning of the support mechanisms more accurate than any other system, such as the drilling of the concrete foundation, mainly because the positioning and verticality of the drills could be questioned.
4. The method chosen to manufacture the base structure makes the process of moving from 3D-design to manufacturing straightfoward. This makes it easy to obtain complex geometries, as is the layout of the different straight, constant curvature and, specially, transition sections of the track.
5. The design allows pre-assembly tasks to be carried out in mechanical workshops.
6. The geometry and extension of the track could be easily modified by adding or removing tables.
7. Maintenance tasks are also easier than in other systems, as the base structure tables could be replaced in the future if necessary.

#### Horizontal projection

**Figure 1:**
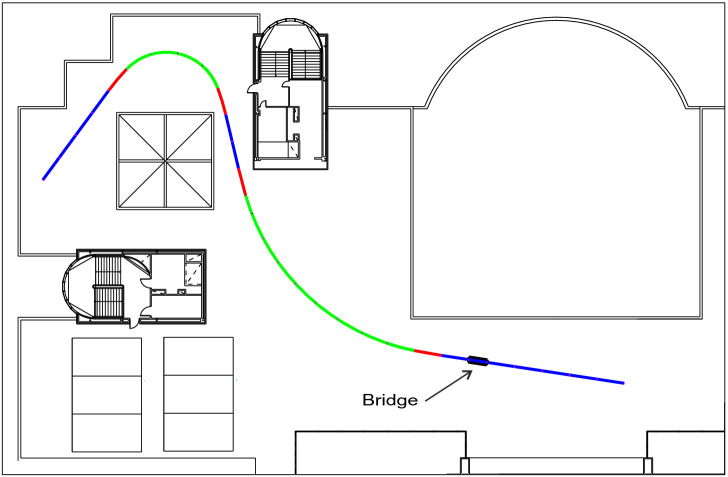
Track horizontal projection. In blue are the 3 straight sections; in red are the 4 transition sections and in green the 2 constant curvature sections. A bridge is located in a straight section.

Given the dimensions of the area of the roof available for the installation of the track, the ideal route that maximizes its length, consists of an open circuit of about 88 meters length constituted by straight, transition, and curved sections, as shown in Fig. 1 with blue, red and green lines respectively, as follows:

1. Straight section of 20 m length. This section includes the bridge-like structure of 2 meters length.
2. Transition of 3 m length.
3. Section of constant curvature of 26 m length and 24 m radius.
4. Transition of 3 m length.
5. Straight section of 6 m length.
6. Transition of 3 m length.
7. Section of constant curvature of 12 m length and 6 m radius.
8. Transition of 3 m length.
9. Straight section of 12 m length.

#### Vertical projection

The track is completely horizontal except for the last 12 meters of straight section, in which a height change of 0.15 m is included. The characteristics of the elevation of the track are specified below:

1. Horizontal section of the first 75.91 m.
2. Section with ascending slope of 3.75$\%$ during 4 m. The total elevation is 0.15 m.
3. Horizontal section of the following 2 m.
4. Section with descending slope of 5$\%$ for 3 m until reaching the initial level of the first section.
5. Horizontal section of 2 m to the end of the track.

To avoid abrupt changes in slope, vertical transition sections are used, with a minimum radius of curvature of 20 m. That is why the radii of the vertical transition sections should be $\ge$ 20 m.

### Support mechanisms

The support mechanisms serve as sleepers and also let the geometry of the track to include track irregularities in the ranges of *D*1, *D*2 and *D*3 according to the standard EN13848^32^. In addition to varying the geometry of the track and allowing long wavelength irregularities to be reproduced, the support mechanisms can correct possible imperfections in the geometrical layout resulting from the manufacture or installation of the track. The support mechanisms (sleepers) are equidistant 0.1 m in the longitudinal direction while in conventional tracks are equidistant around 0.6 m. The distance between the support mechanisms is greater than what corresponds to the length scaling factor of 1/11.3 in order to facilitate the access and to vary the geometry easily.

**Figure 2:**
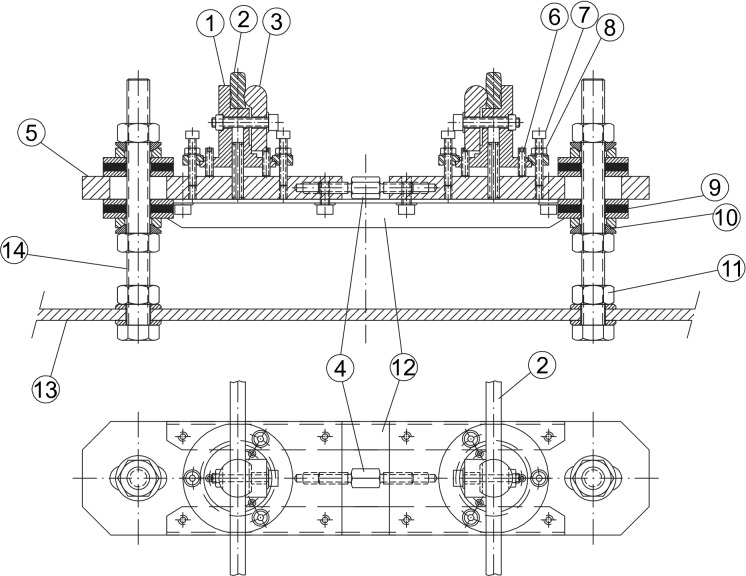
Support mechanisms design.

The support mechanisms are described below, each part being indicated by a number in parentheses corresponding to the number shown in Fig. 2.

**Figure 3:**
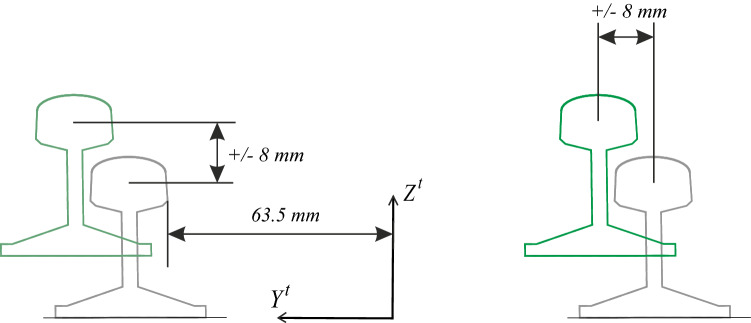
Displacement of the rails.

**Figure 4:**
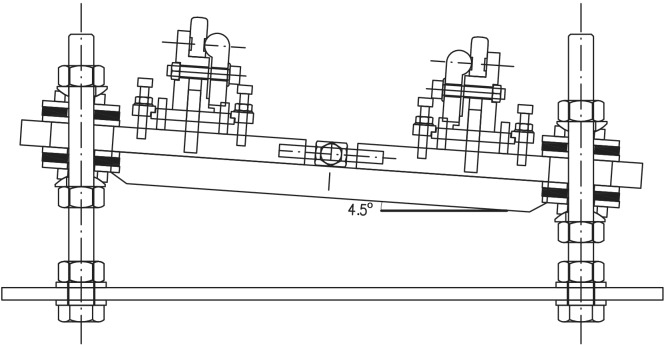
Support mechanism designed with maximum cant angle.

According to Fig. 2, two screws (14) are rigidly joined to the surface of the table (13). Two strong plates (5) are supported on the studs joined by a pair of angles (12). The distance between the strong plates (5), which will determine the track gauge, can be modified thanks to the tensioner (4). The support mechanisms of the rails are on the plates. A central body (1) receives the rail (2), which is fixed thanks to the clamp (3). Adjustment studs (6) allow the central body position to be adjusted ± 5 mm vertically. The screw (7) acts on the flange (8), fixing the central body. Both nuts (11) for fixing the plates on the screws (14) fit perfectly thanks to the interposed bevel washers (10). Two neoprene rubber washers with 60 Shore hardness, 37 mm diameter and 4 mm thickness (9) materialise the desired flexibility conditions for the support of the rails. From the preceding description, it is deduced that the mechanisms have the capacity to adapt the rails to the required geometry, as described next:

1. The mechanisms allow a movement of each rail independently in the *Y*-lateral direction of ± 0.008 m and in *Z* of ± 0.008 m (see Fig. 3).
2. The mechanisms are designed so that a height difference between the two rails of 10 mm in the *Z* direction can be allowed, so that a maximum cant angle of 4.5$^\circ$ can be reproduced (see Fig. 4).
3. The free rotation of the central bodies in vertical *Z* axis, and therefore of the rail, is allowed. This feature is especially useful in curves and transition sections.
4. It allows a rail rotation of ±3$^\circ$ in *Y* axis, which is useful in slopes.

**Figure 5:**
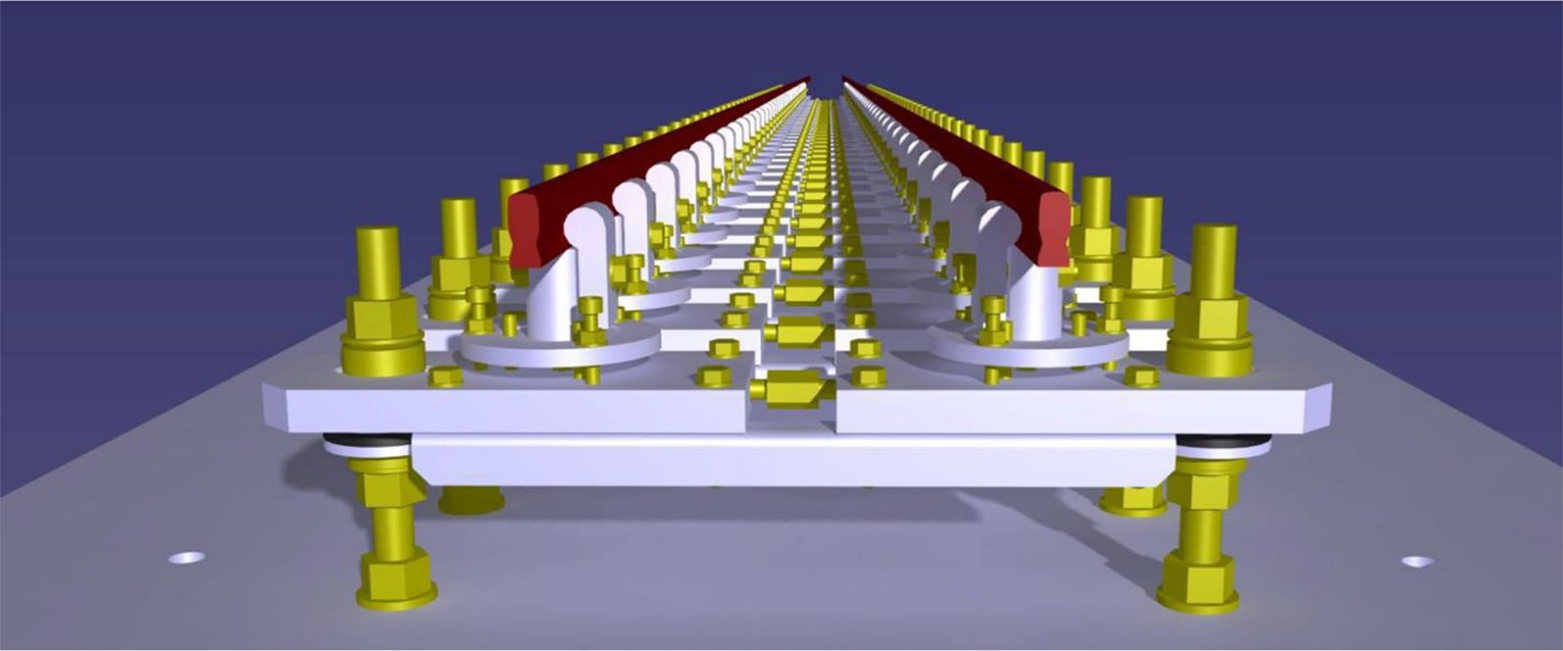
3D render view of the designed mechanisms.

The appearance that the mechanisms on the base structure will have is shown as a render image in Fig. 5.

### Rails

**Figure 6:**
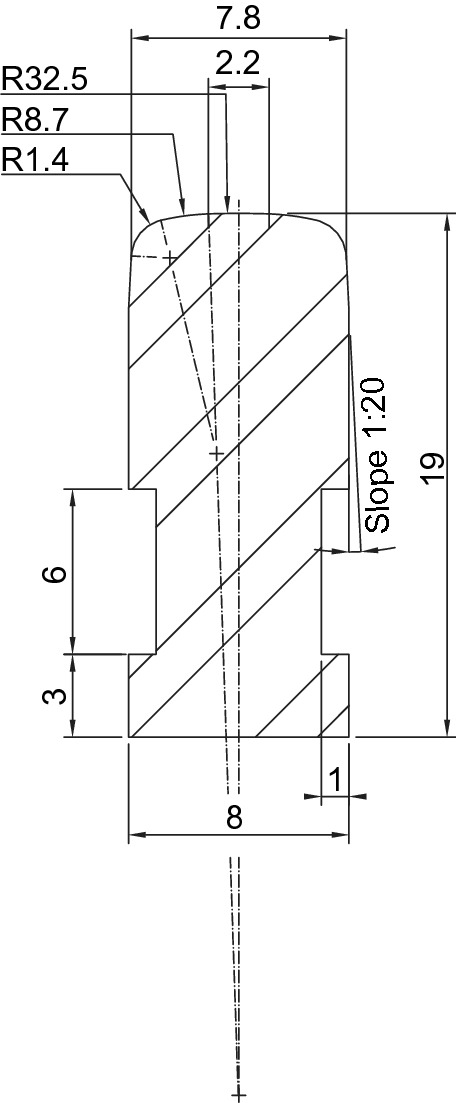
Rail cross-section.

**Figure 7:**
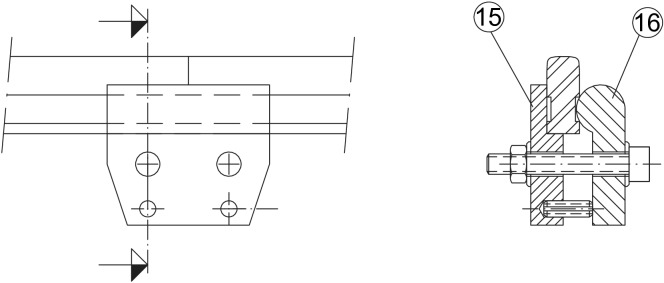
Cross-section of the rail to rail clamp.

The rails cross-section are obtained by machining sectional 8 x 20 mm stainless steel bars. It is characterised by having the railhead equal to the normalised UIC60 profile and a total width of 8 mm. The height (19 mm after machined) is not scaled since only the railhead profile keeps contact with the wheels. The rail cross-section is shown on Fig. 6. A double lateral groove which serves as support for the clamps can be observed, which maintains the position and alignment of the rails. There are two kinds of clamps:

1. Rail to support mechanism clamp, shown in Fig. 2 with numbers (1) and (3).
2. Rail to rail clamp. The configuration of the track is based on independent rail sections of about 3 m length which are mechanically joined by clamps, shown in Fig. 7 with numbers (15) and (16).

The material used for the rails is AISI 304 stainless steel, milled in a CNC machine. To join consecutive rails, their ends are bevel-cut at 45$^\circ$, so that the discontinuities between consecutive rails are minimized. Also, two expansion joints have been considerated as sliding joints, with a rigid connection in one of the sections to be joined and a sliding one in the other. In this context, the expansion joins will be placed along straight track sections to minimize misalignments.

### Installed scaled track

**Figure 8:**
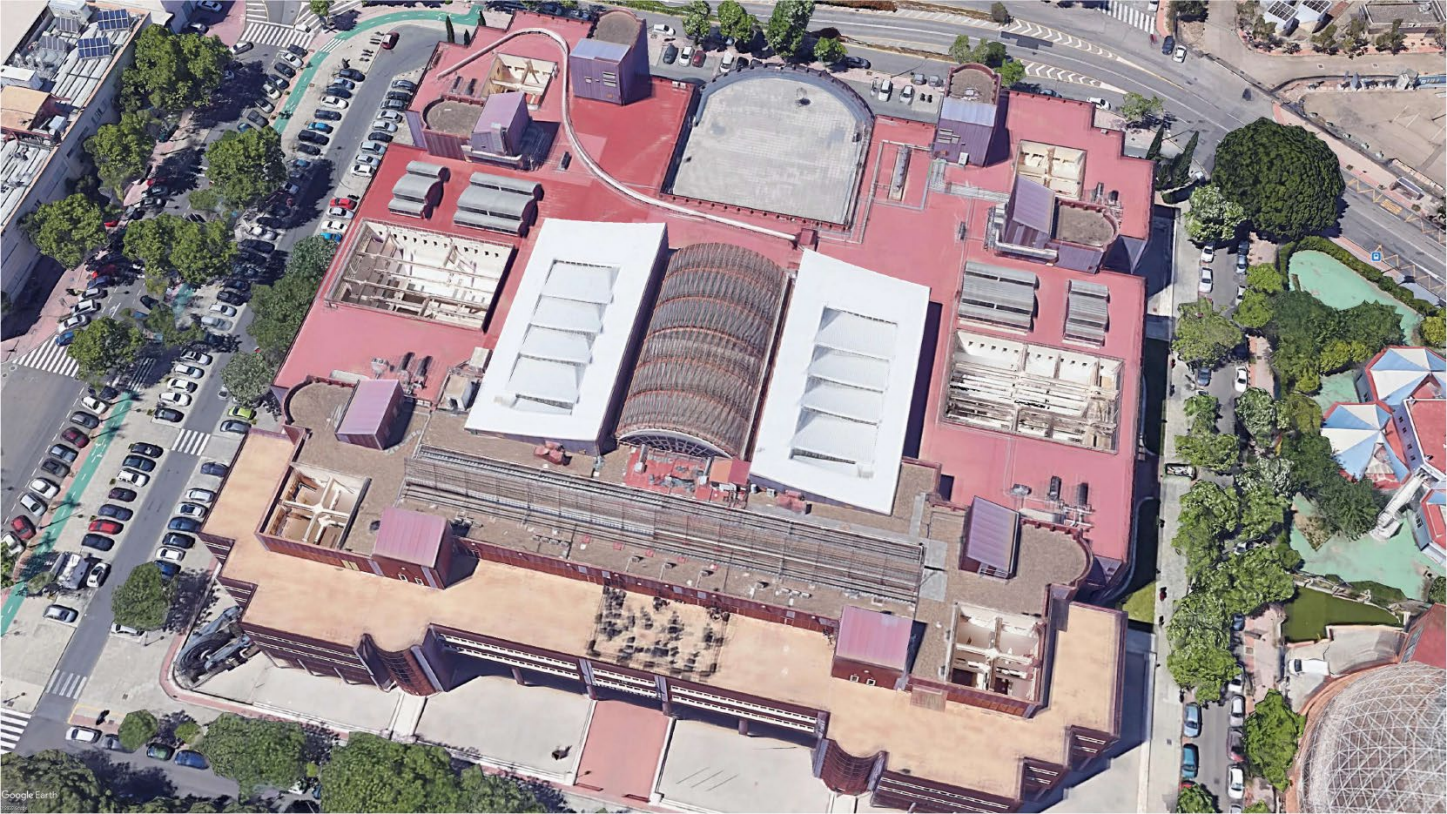
Aereal view of the track installed at the upper left corner of the roof of the School of Engineering of the University of Seville (obtained from Google Maps).

The scaled track is shown in the upper left corner of the building in Fig. 8, which is an aereal view.

**Figure 9:**
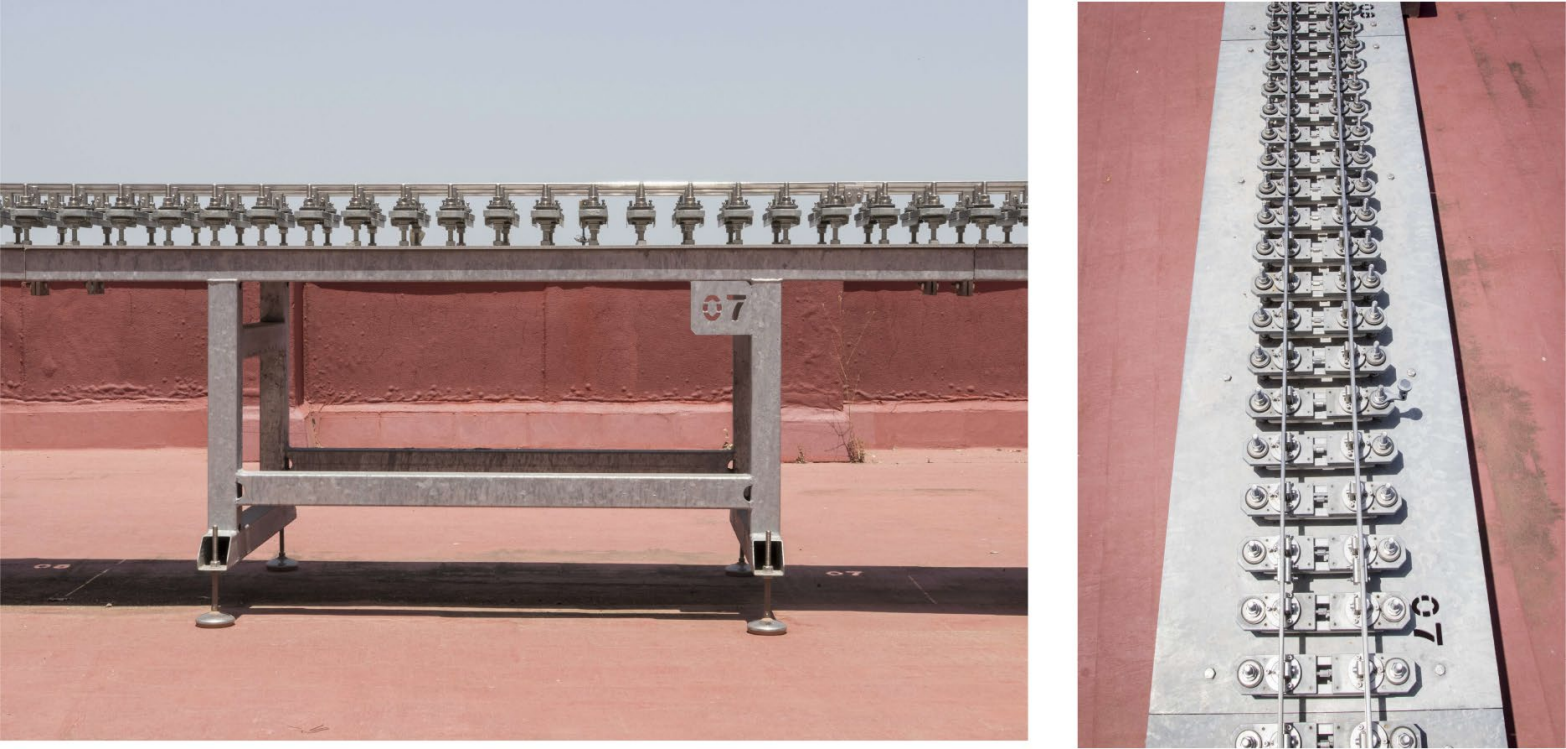
Table support structure: elevation view (left); horizontal view (right). Photographer: Ludivine Mimar.

**Figure 10:**
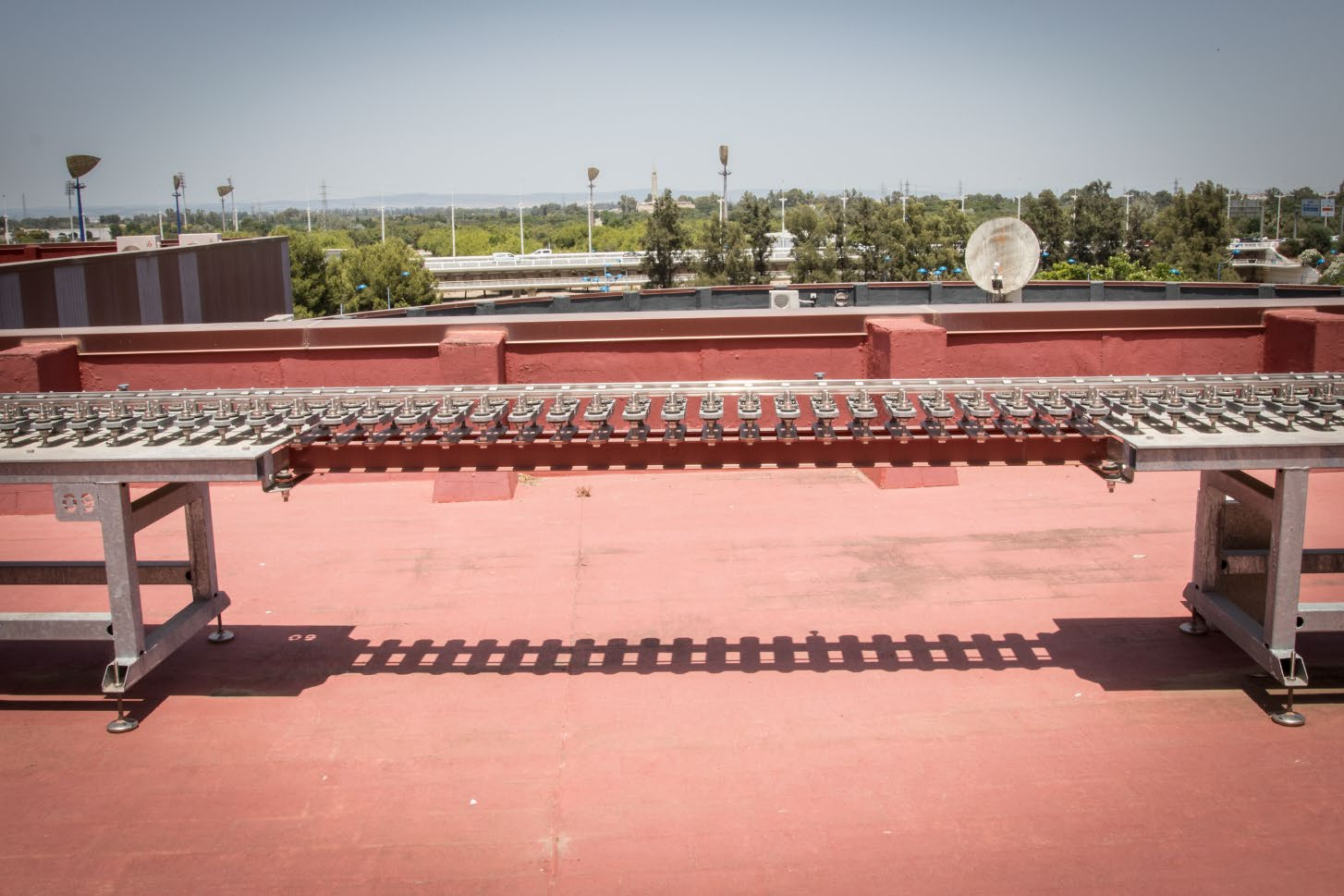
View of the 2m-length bridge. Photographer: Ludivine Mimar.

**Figure 11:**
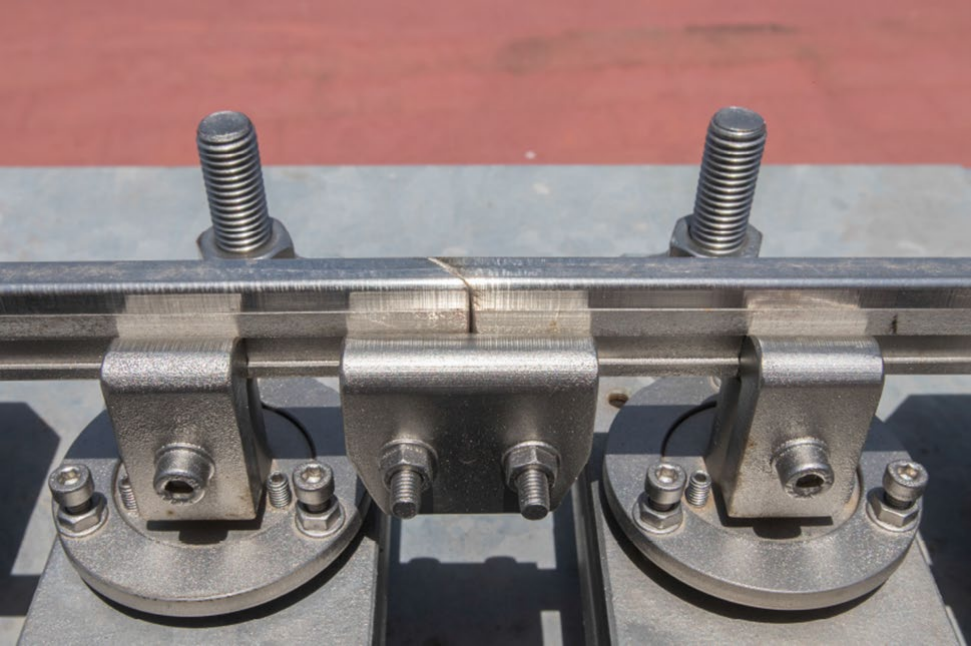
Rail to rail clamp. Photographer: Ludivine Mimar.

**Figure 12:**
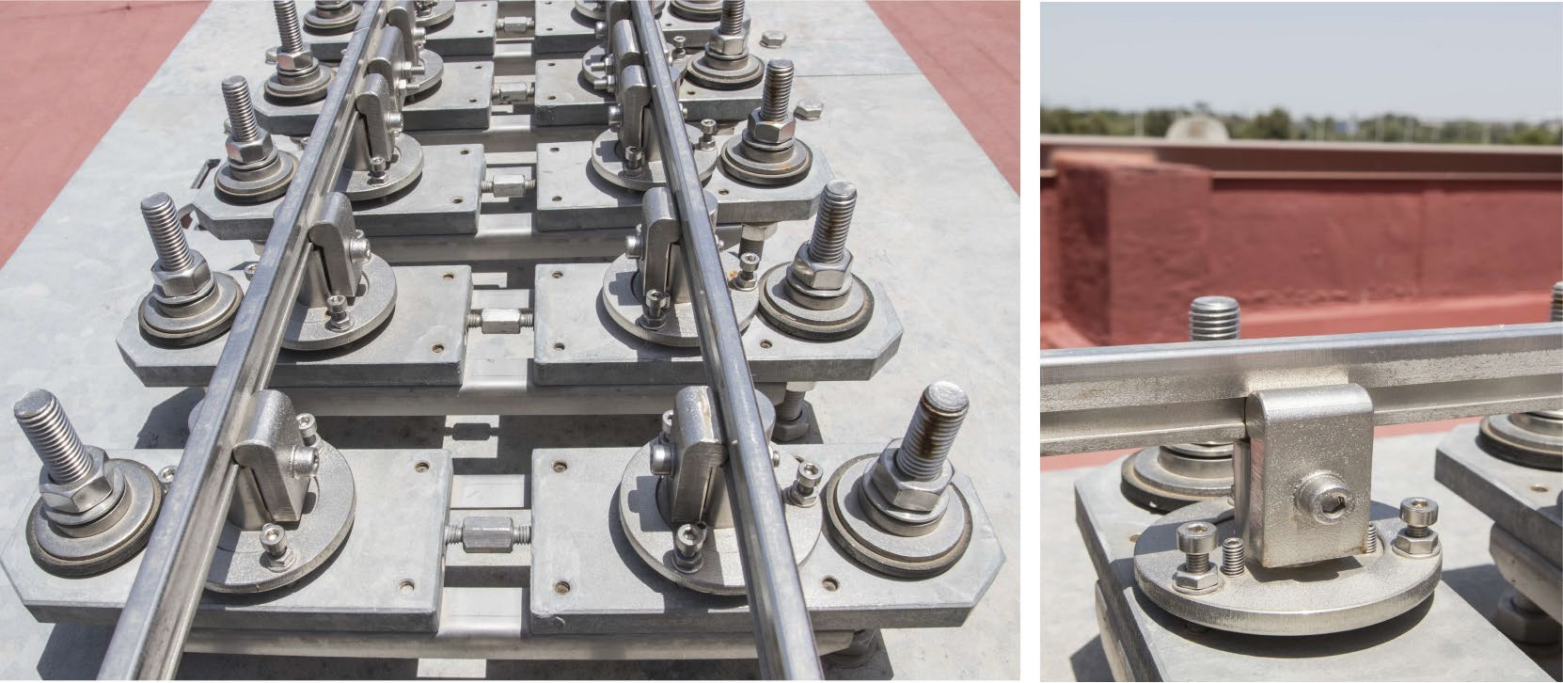
Support mechanisms (left) and rail to support mechanism clamp (right). Photographer: Ludivine Mimar.

**Figure 13:**
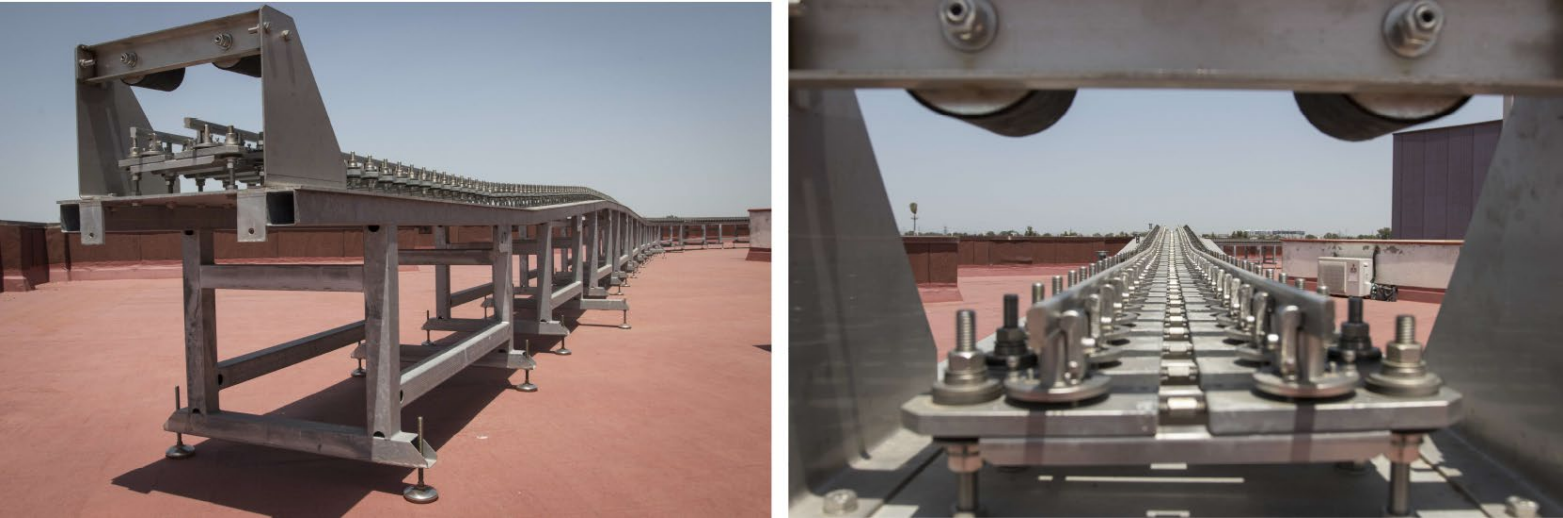
Structure with ascending and descending slopes (left) and buffer stops at the end of the track (right). Photographer: Ludivine Mimar.

**Figure 14:**
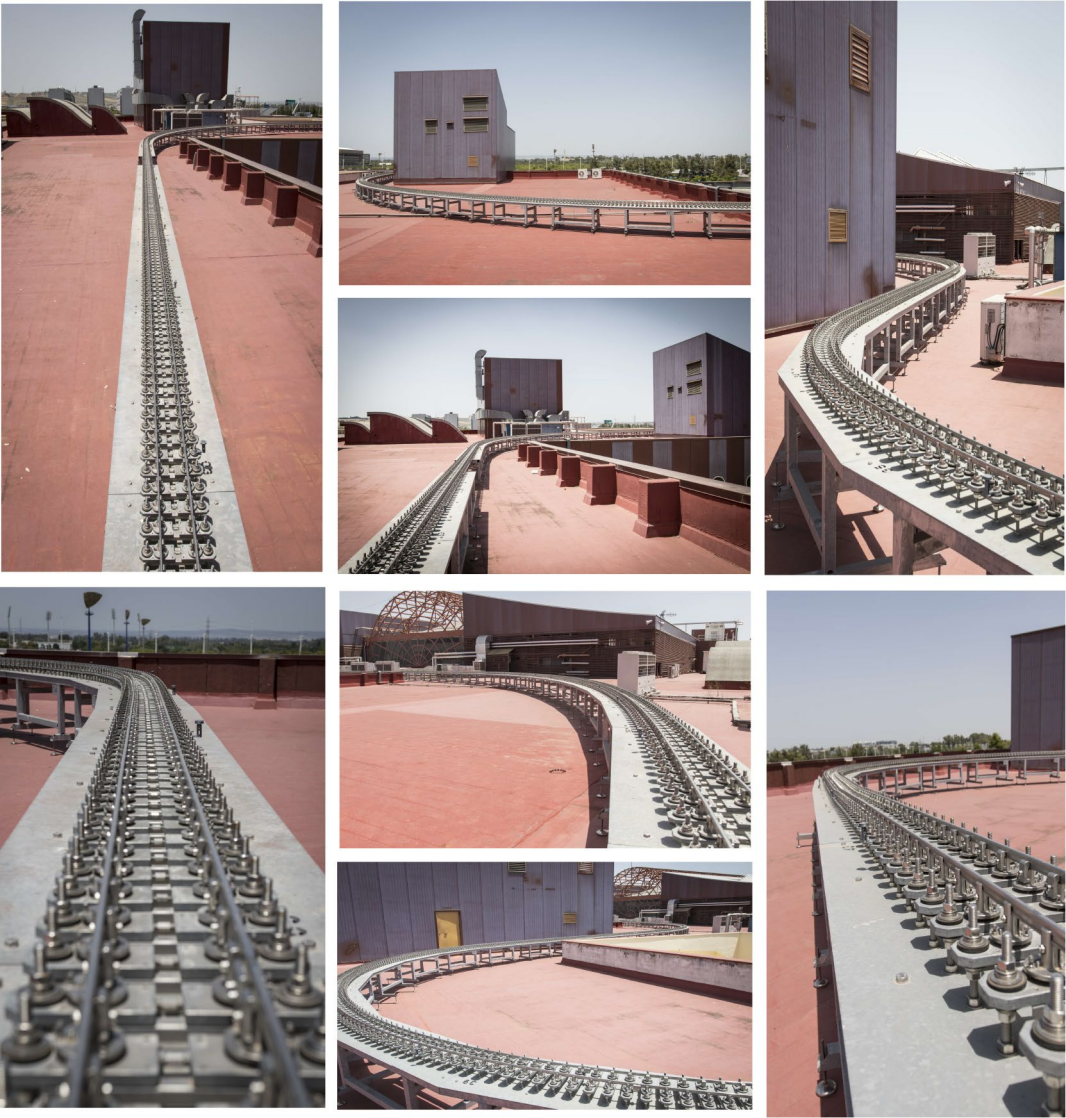
Different views of the track. Photographer: Ludivine Mimar.

Figure 9 shows one of the tables that constitutes the base structure, it is the table no. 7 that belongs to a straight track section. The bridge is shown in Fig. 10, whose base structure is formed by 2 laminated IPE80 bars. In Fig. 11, a detail of the joint between consecutive rails can be seen. Figure 12 shows the mechanisms that fasten the rails and a detailed view of them. Figure 13 shows the end of the track where the sections with vertical slope are located. Finally, Fig. 14 shows different views of the track, where straight, curved and transition sections can be distinguished.

## Quality controls performed on the scaled track

Certain quality controls were carried out after the installation of the scaled track. (1) A study of the response of the track due to vertical and lateral loads, and (2) a track geometry measurement with a topographic equipment to know the real geometry of the track. The obtained results are shown in the following subsections.

### Response to loads

**Figure 15:**
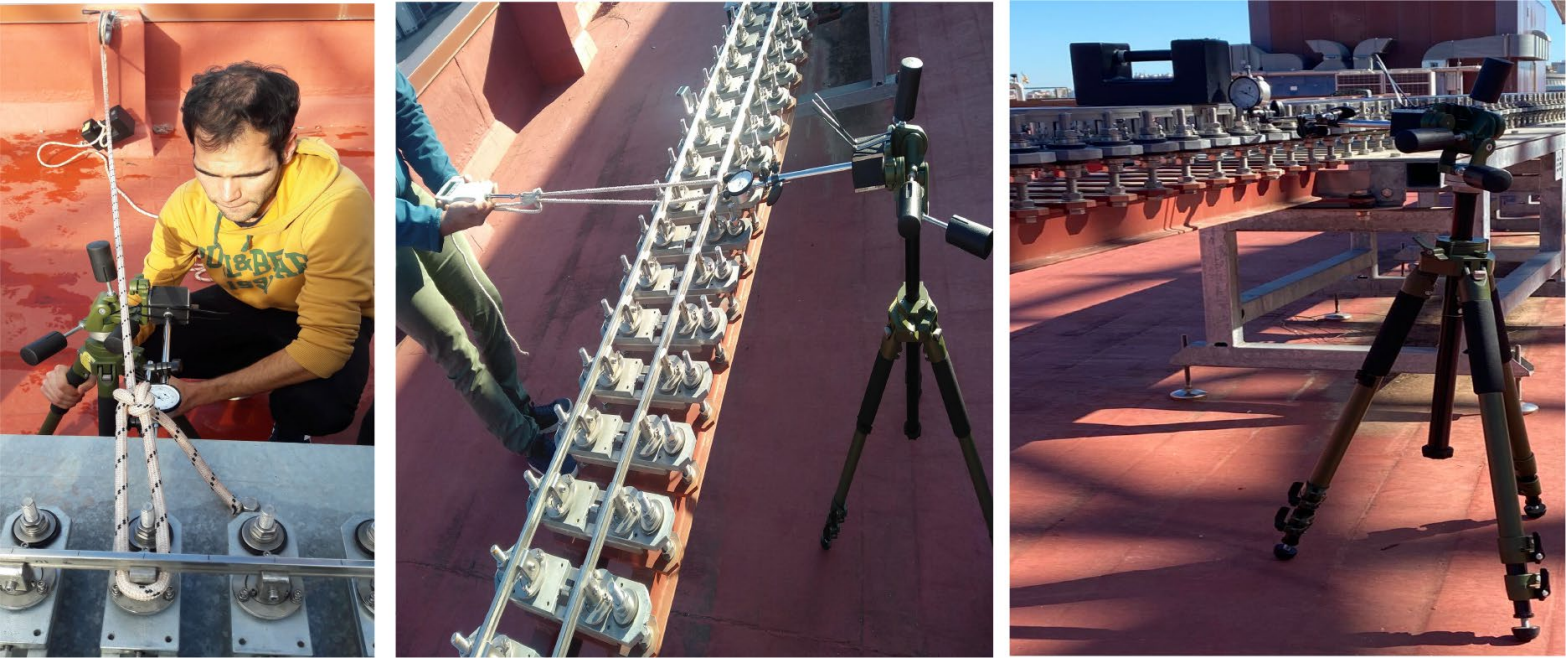
Measurement of track response to concentrated loads. Lateral loading with pulley, mass and dial gauge (left), lateral loading with dynamometer and dial gauge (center), and vertical loading with mass and dial gauge (right).

**Figure 16:**
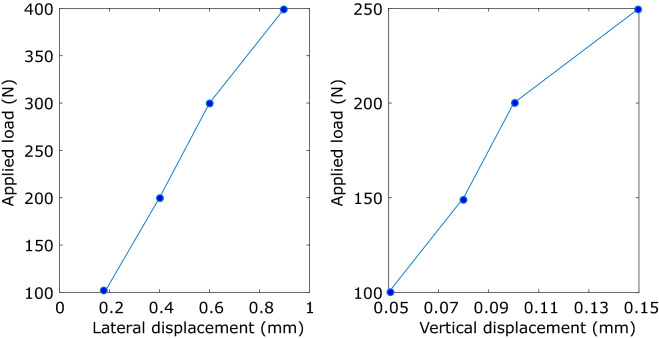
Mechanism support lateral (left) and vertical (right) displacement measured at the middle of the bridge when different loads are applied.

Different types of tests have been carried out to measure the displacement of the structure when lateral and vertical loads are applied. The deflection of the structure is measured with a dial gauge supported on a tripod (see Fig. 15). The description of these tests and the obtained results are explained next:

1. Test 1: response to a lateral force. Using a pulley anchored to a fixed point and a rope, a load of 200 N is applied at different points of the circuit in the lateral direction. The results obtained are displayed in Table 1. The following nomenclature is used to identify the measurement points: $XX-YYY-Z$, where *XX* corresponds to the number that identifies the structure (see number 07, for example in Fig. 9), *YYY* with the distance in mm from the beginning of the corresponding structure, and *Z* can be *R* for the right rail or *L* for the left rail^41^.
2. Test 2: response to a vertical force. A mass of 20 kg was supported on the rails over the support mechanisms, and the vertical displacement (in particular, the part numbered (5) in Fig. 2) were measured. The obtained response is shown in Table 2. The following nomenclature is used to identify the measurement points: $XX-YYY$, where *XX* corresponds to the number that identifies the structure, or to the letters *BR* that indicate the bridge, and *YYY* with the distance in mm from the beginning of the corresponding part of the structure^41^.
3. Test 3: lateral load on the bridge. Different loads were applied in the middle of the 2-m length bridge and measured with a dynamometer (100, 200, 300 and 400 N). The results obtained are displayed in Fig. 16 (left) where it can be seen that the lateral displacement linearly increases with the load.
4. Test 4: vertical load on the bridge. Different masses were placed in the middle on the bridge over the support mechanisms, and the vertical displacement (in particular, the part numbered (5) in Fig. 2) were measured. Results are shown in Fig. 16 (right) where it is shown that the vertical displacement also linearly increases with the load.

With the different applied experiments, it was observed that the structure does not suffer permanent deformation in lateral and vertical directions. It is also observed that the bridge, which is the least stiff part of the track, experiences a linear trend behaviour in its middle point. Test results support that the rigid track assumption is reasonable. That conclusion is taken after considering the forces and displacements that would occur when a 70 kg vehicle runs on the small radius curve (*R* = 6 m) with the relatively high velocity of 3 m/s. In that scenario, the centrifugal force is 105 N and the lateral displacement of the vehicle is 3.2 mm if the track is assumed rigid. This value of the lateral displacement is the wheel-rail clearance. If the track is flexible, using the stiffness obtained in the experimental tests, the increment in lateral diplacement due to the track flexibility would be 0.2 mm (see Fig. 16), that is smaller than one tenth of the “rigid track displacement.”

**Table 1:** Response of the track to a 200 N lateral load.

Measurement point	Displacement (mm)
04-100-R (rail)	0.15
04-100-R (support)	0.15
13-100-R (rail)	0.02
13-100-R (support)	0.02
37-010-R (rail)	0.06
37-010-R (support)	0.06

**Table 2:** Track response to a vertical load of 200 N applied in different locations.

Measurement point	Displacement (mm)
38-090	0.03
47-010	0.06
03-030	0.05
08-170	0.06
*BR*-110	0.10
*BR*-170	0.09
16-030	0.03
18-155	0.04

### Topographic measurements

To know the real geometry of the installed track, the absolute position of a series of points located on the head of both rails and separated 50 mm is measured. The selected distance allows to measure an irregularity with a minimum wavelength up to 100 mm. The position is measured with a total station (brand TRIMBLE, model VX, capable of measuring angles to one arc-second). For each measured point, the lateral and vertical distance to the theoretical position that each point should have according to the initial design are calculated, in order to determine the initial irregularities that the track has. This experimental procedure is described in^34^.

The following three criteria are established to evaluate the quality of the manufacturing and installation of the track:

1. A global criterion: The absolute distance from a point to its position in the design geometry should not exceed 0.05 m. This value was selected so that there is a clearance between the track and the surrounding building.
2. An irregularity criterion: Upon application to the irregularities obtained from topographic measurements of a 4th order band-pass Butterworth filter, with wavelengths $\lambda _{min}$ = 0.3m and $\lambda _{max}$ = 2.5 m (scaled *D*1 level according to EN13848-5^32^), the maximum lateral and vertical distance to the design points should not exceed 2 mm.
3. Gauge criterion: the track gauge irregularity, measured with the absolute distance between the internal faces of the rails, should not be less than -1 mm nor greater than 2.5 mm.

The criterion established for the track gauge is based on the limits established by UNE-EN 13848-5^32^. In this standard, for speeds below 120 km/h, isolated defects of the track gauge range from $-$ 11 mm up to 35 mm of the nominal value where 1.435 m track gauge is considered as nominal value in the standard. Considering that the nominal width is 0.127 m in the scaled track, for a scaled speed lower than 2.95 m/s, some limits of $-$ 0.98 mm and +3.1 mm may be permitted.

**Figure 17:**
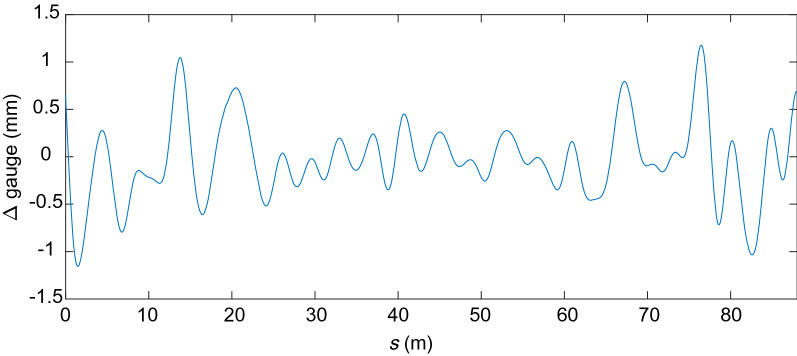
Filtered scaled track gauge deviation.

**Figure 18:**
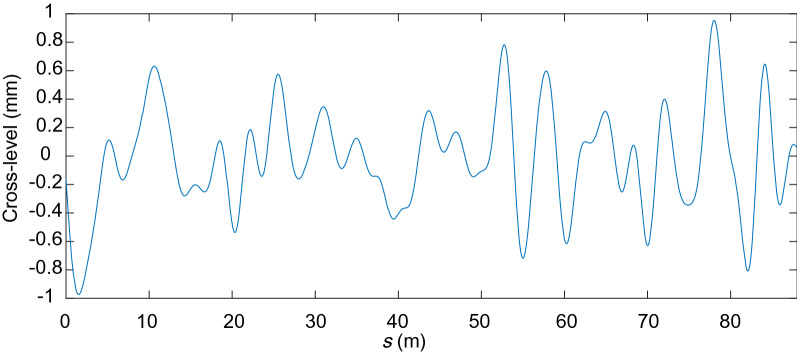
Filtered scaled track cross-level irregularity.

**Figure 19:**
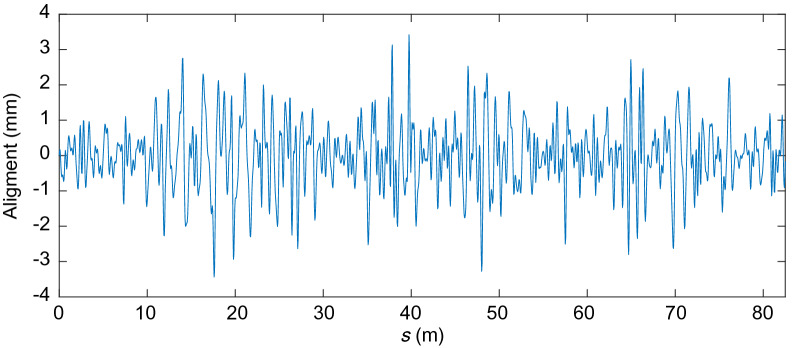
Filtered scaled track alignment irregularity.

**Figure 20:**
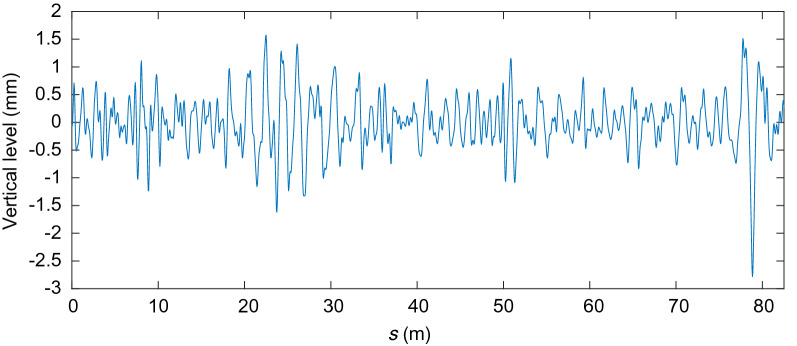
Filtered scaled track vertical profile irregularity.

Figures 17, 18, 19 and 20 show the irregularities obtained after applying the band-pass filter: gauge deviation, cross-level, alignment and vertical profile, respectively. It can be seen that the measurements are considered within the allowed range The irregularities measured to check the quality of the installation of the track are the static ones. However, measuring the track geometry using an automated recording vehicle has also been applied in this scaled track, and the comparison between the static and dynamic measurement can be found in^42^.

## Summary and conclusions

This article presents the process of design, manufacture and installation of a scaled railroad track that allows to introduce track irregularities to its reference geometry such as gauge variation, cross-level, alignment and vertical profile. The main advantage of the scaled track presented in this article is that it allows the development of different methods that can be extrapolated to conventional systems. Some examples of these extrapolatable methods which are developed using this scaled track, are the methods that monitor the geometry and detect irregularities that will improve the maintenance of railway tracks, and methods that allow to study the effect of the irregularities in the dynamics of the vehicles. The track is not designed to directly extrapolate the dynamics effects obtained at the scaled size to the full-scaled one. The scaled track allows an easy access to experimental trials under controlled conditions that are not easily accessible on conventional tracks. It is based on design specifications and, based on them, the design carried out is explained. The results obtained after the manufacture and installation of the track are shown in this article. Some studies that can be carried out with the track have been specified. However, it allows the use of more different studies, such as the study of the effect of different fasteners, the introduction of specific short-wavelength surface rail irregularities or the addition of track segments containing switches and crossings. In those cases, simple but new elements designs are required, which shows the broad range of applications of this facility. Various tests are carried out to check the quality of the track; they include load tests in vertical and lateral direction, and the measurement of the geometry of both rails. The load tests have shown that the response of the base structure and the support mechanisms will not undergo permanent deformations for the applied loads and that the stiffness of the bridge, which is the most flexible part of the track, has a linear trend behaviour. Also, test results support that the rigid track assumption is reasonable. In addition, it is shown that the irregularities measured at the track meet the specified requirements.

### Human participant

Informed consent was obtained from all participants for the publication of identifying images in an online open-access publication.

## Data Availability

Accession codes: Correspondence and requests for materials should be addressed to R.C.

## References

[1] Glickenstein H (2007). SNCF and Alstom set new world speed record. IEEE Veh. Technol. Mag..

[2] Weidemann C (2010). State-of-the-art railway vehicle design with multi-body simulation. J. Mech. Syst. Transp. Logist..

[3] Kortüm, W. & Sharp, R. S. The IAVSD review of multibody computer codes for vehicle system dynamics. In *Proceedings of the third SME Symposium on transportation systems* (ed. Pombo, J.) (Anaheim, ASME Winter Anual Meeting, Anaheim, CA, 1992).

[4] Bergander, B. & Kunnes, W. ERRI B176/DT 290: B176/3 Benchmark problem, results and assessment. Technical report, European Rail Research Institute (1993).

[5] Kortüm, W. & Sharp, R. S. Multibody computer codes in vehicle system dynamics. Swets & Zeitlinger (1993).

[6] Iwnicki, S. The Manchester benchmarks for rail vehicle simulation. Swets and Zeitlinger (1999).

[7] Bezin Y (2021). Multibody simulation benchmark for dynamic vehicle-track interaction in switches and crossings: results and method statements. Veh. Syst. Dyn..

[8] Jaschinski A, Chollet H, Iwnicki S, Wickens A, Würzen JV (1999). The application of roller rigs to railway vehicle dynamics. Veh. Syst. Dyn..

[9] Meymand, S. *State of the art roller rig for precise evaluation of wheel-rail contact mechanics and dynamics*. PhD Thesis, Faculty of the Virginia Polytechnic Institute and State University, Blacksburg, VA (2015).

[10] Allen, P. *et al.* Roller rigs. In *Handbook of Railway Vehicle Dynamics* (eds. Iwnicki, S. *et al.*), chap. 19, 761–823 (2020).

[11] Myamlin S, Kalivoda J, Neduzha L (2017). Testing of railway vehicles using roller rigs. Procedia Eng..

[12] The Secretary of Transportation. The Tenth and Final Report on the High Speed Ground Transportation Act of 1965. Tech. Rep., U.S. Department of Transportation (1977).

[13] Transportation Technology Center, Inc., Laboratory facility in Pueblo, Colorado. (Accessed 2 March 2022 $$<$$https://www.ttci.tech/ttci-facility-transition$$>$$).

[14] MHI. Introduction of the multipurpose integrated highly-advanced railway applications (MIHARA) test center. Tech. Rep., Mitsubishi Heavy Industries Technical Review, 52(1) (2015).

[15] Gipson G, Yeigh B (2005). Scaling issues related to modeling of railroad car damage i-derailment, plastic deformation, rupture, and impact. Math. Comput. Model..

[16] Iwnicki S (2006). Handbook of Railway Vehicle Dynamics.

[17] Liu B, Bruni S (2015). A method for testing railway wheel sets on a full-scale roller rig. Veh. Syst. Dyn..

[18] Matsudaira T, Matsui N, Arai S, Yokose K (1969). Problems on hunting of railway vehicles on test stand. J. Eng. Ind..

[19] Elkins J, Wilson N (1986). Train resistance measurements using a roller rig. Veh. Syst. Dyn. Int. J. Veh. Mech. Mobil..

[20] Zhang, W., Dai, H. Shen, Z. & Zeng, J. In * Handbook of Railway Vehicle Dynamics* (ed. Iwnicki, S.), chap. 14, 457–506 (2006).

[21] Michitsuji Y, Suda Y (2006). Running performance of power-steering railway bogie with independently rotating wheels. Veh. Syst. Dyn..

[22] Lin, S., Tomimatsu, D., Nishimura, K., Yabuno, H. & Suda, Y. Stabilization of single axle truck hunting motion using a gyroscopic damper with a gravitational restoring mechanism. In *Proceedings of the First International Conference on Railway Technology: Research, Development and Maintenance* Vol. 1 (ed. Pombo, J.) 1–8 (Civil-Comp Press, Stirlingshire, Scotland, 2012).

[23] Suda Y, Wang W, Nishina M, Lin S, Michitsuji Y (2012). Self-steering ability of the proposed new concept of independently rotating wheels using inverse tread conicity. Veh. Syst. Dyn..

[24] Kim M-S, Park J-H, You W-H (2008). Construction of active steering system of a scaled railway vehicle. Int. J. Syst. Appl. Eng. Dev..

[25] Kim M-S, Hur H-M (2009). Application of braking/traction control systems to the scaled steering testbed in the railway vehicle. WSEAS Trans. Syst. Control.

[26] Zhu J, Thompson D, Jones CJC (2011). On the effect of unsupported sleepers on the dynamic behaviour of a railway track. Veh. Syst. Dyn..

[27] Naeimi M (2014). Substantial fatigue similarity of a new small-scale test rig to actual wheel-rail system. World Acad. Sci. Eng. Technol. Int. J. Mech. Aerosp. Ind. Mechatron. Eng..

[28] Naeimi M, Li Z, Dollevoet R (2014). Scaling strategy of a new experimental rig for wheel-rail contact. World Acad. Sci. Eng. Technol. Int. J. Mech. Aerosp. Ind. Mechatron. Eng..

[29] Naeimi M, Li Z, Petrov R, Sietsma J, Dollevoet R (2018). Development of a new downscale setup for wheel-rail contact experiments under impact loading conditions. World Acad. Sci. Eng. Technol. Int. J. Mech. Aerosp. Ind. Mechatron. Eng..

[30] Standard. UIC 518. Railway applications. Track. Track geometry quality. Part 1: Characterisation of track geometry. British Standards Institution, London (2003).

[31] Standard. UNE-EN 13231-1. Railway applications. Track. Track geometry quality. Part 1: Characterisation of track geometry. British Standards Institution, London (2003).

[32] Standard. UNE EN-13848-5:2009. Railway applications. Track. Track geometry quality. Part 5: Geometry quality levels. AENOR (2009).

[33] Allen, P. Scale Testing. In *Handbook of Railway Vehicle Dynamics* (ed. Iwnicki, S.), chap. 15, 507–526 (2006).

[34] Aceituno J, Chamorro R, Muñoz S, Escalona J (2019). An alternative procedure to measure railroad track irregularities. Application to a scaled track. Measurement.

[35] Aceituno JF, Chamorro R, García-Vallejo D, Escalona JL (2017). On the design of a scaled railroad vehicle for the validation of computational models. Mech. Mach. Theory.

[36] Escalona J, Urda P, Muñoz S (2021). A track geometry measuring system based on multibody kinematics, inertial sensors and computer vision. Sensors.

[37] Gullers P, Andersson L, Lundén R (2008). High-frequency vertical wheel-rail contact forces-field measurements and influence of track irregularities. Wear.

[38] Urda P, Muñoz S, Aceituno J, Escalona J (2020). Wheel-rail contact force measurement using strain gauges and distance lasers on a scaled railway vehicle. Mech. Syst. Signal Process..

[39] De la Viña Reina FJ (2014). Diseño de una vía de tren a escala y de mecanismos que simulen irregularidades en ella.

[40] Standard. UNE-EN ISO 1461:2010. Hot dip galvanized coatings on fabricated iron and steel articles—specifications and test methods (2010). ISO 1461:2009.

[41] Urda, P., Muñoz, S. & Chamorro, R. Internal Report (Load application. Tech. Rep., University of Seville, Verification of the minimum requirement, 2017).

[42] Urda P, Aceituno J, Muñoz S, Escalona J (2021). Measurement of railroad track irregularities using an automated recording vehicle. Measurement.

